# Impact of Chorionic Somatomammotropin In Vivo RNA Interference Phenotype on Uteroplacental Expression of the IGF Axis

**DOI:** 10.3390/life13061261

**Published:** 2023-05-26

**Authors:** Taylor K. Hord, Amelia R. Tanner, Victoria C. Kennedy, Cameron S. Lynch, Quinton A. Winger, Paul J. Rozance, Russell V. Anthony

**Affiliations:** 1 College of Veterinary Medicine, Colorado State University, Fort Collins, CO 80523, USA; 2 Anschutz Medical Campus, University of Colorado School of Medicine, Aurora, CO 80045, USA

**Keywords:** placenta, CSH, RNA interference, fetal growth restriction, IGF1, IGF2, IGFBP

## Abstract

While fetal growth is dependent on many factors, optimal placental function is a prerequisite for a normal pregnancy outcome. The majority of fetal growth-restricted (FGR) pregnancies result from placental insufficiency (PI). The insulin-like growth factors (IGF1 and IGF2) stimulate fetal growth and placental development and function. Previously, we demonstrated that in vivo RNA interference (RNAi) of the placental hormone, chorionic somatomammotropin (CSH), resulted in two phenotypes. One phenotype exhibits significant placental and fetal growth restriction (PI-FGR), impaired placental nutrient transport, and significant reductions in umbilical insulin and IGF1. The other phenotype does not exhibit statistically significant changes in placental or fetal growth (non-FGR). It was our objective to further characterize these two phenotypes by determining the impact of CSH RNAi on the placental (maternal caruncle and fetal cotyledon) expression of the IGF axis. The trophectoderm of hatched blastocysts (9 days of gestation, dGA) were infected with a lentivirus expressing either a non-targeting sequence (NTS RNAi) control or CSH-specific shRNA (CSH RNAi) prior to embryo transfer into synchronized recipient ewes. At ≈125 dGA, pregnancies were fitted with vascular catheters to undergo steady-state metabolic studies. Nutrient uptakes were determined, and tissues were harvested at necropsy. In both CSH RNAi non-FGR and PI-FGR pregnancies, uterine blood flow was significantly reduced (*p* ≤ 0.05), while umbilical blood flow (*p* ≤ 0.01), both uterine and umbilical glucose and oxygen uptakes (*p* ≤ 0.05), and umbilical concentrations of insulin and IGF1 (*p* ≤ 0.05) were reduced in CSH RNAi PI-FGR pregnancies. Fetal cotyledon *IGF1* mRNA concentration was reduced (*p* ≤ 0.05) in CSH RNAi PI-FGR pregnancies, whereas neither *IGF1* nor *IGF2* mRNA concentrations were impacted in the maternal caruncles, and either placental tissue in the non-FGR pregnancies. Fetal cotyledon *IGF1R* and *IGF2R* mRNA concentrations were not impacted for either phenotype, yet *IGF2R* was increased (*p* ≤ 0.01) in the maternal caruncles of CSH RNAi PI-FGR pregnancies. For the IGF binding proteins (IGFBP1, IGFBP2, IGFBP3), only *IGFBP2* mRNA concentrations were impacted, with elevated *IGFBP2* mRNA in both the fetal cotyledon (*p* ≤ 0.01) and maternal caruncle (*p* = 0.08) of CSH RNAi non-FGR pregnancies. These data support the importance of IGF1 in placental growth and function but may also implicate IGFBP2 in salvaging placental growth in non-FGR pregnancies.

## 1. Introduction

Fetal growth restriction (FGR), a major pregnancy complication, is often characterized by abnormal placental development and function. Since the placenta is the interface between maternal and fetal circulation, it provides the means necessary for the exchange of nutrients and gases with the fetus. Consequently, abnormal placental development can hinder nutrient transfer to the fetus [1]. Insulin-like growth factors (IGFs) are known for their involvement and critical role in promoting the growth of the fetus and placenta during gestation [2], and altered expression of the IGFs is associated with growth deficiency in both embryonic growth and placental growth [3,4]. The IGF axis is comprised of insulin-like growth factor 1 (IGF1), IGF2, the type 1 IGF receptor (IGF1R), IGF2R, and 6 IGF-binding proteins (IGFBP1-6; [5]), many of which have been implicated in the regulation of fetal growth and development. The importance of both IGF1 and IGF2 for placental development was clearly demonstrated by placental-specific gene ablations, which resulted in fetal growth restriction and placental growth restriction [6,7]. In humans, reduced fetal plasma IGF1 concentration is correlated with reduced birth weight [8], and IGF1 concentrations are reduced in FGR pregnancies [9]. Both IGF1 and IGF2 interact with IGF1R to elicit functional cellular responses [2], whereas IGF2R acts as a clearance receptor for IGF2 [3,10]. Mouse models carrying null mutations in Igf1r led to embryonic and placental growth deficiency [3], and an Igf2r knockout mouse model resulted in fetal overgrowth and perinatal lethality [11]. The IGF binding proteins regulate IGF availability and IGF biological activity, although gene ablation studies have yet to demonstrate the necessity of the IGFBP’s in placental and fetal growth. However, it is known that IGFBP1, 2, and 3 are expressed by the placenta and have the potential to either potentiate or inhibit the activity of the IGFs [4].

It has long been hypothesized that chorionic somatomammotropin/placental lactogen (CSH/PL) acts as a key regulator for fetal growth, partly through its potential stimulation of maternal and fetal IGF production [12]. Recently lentiviral-mediated RNA interference (RNAi) induced CSH-deficient pregnancies have been reported [13,14,15], which result in fetal and placental growth restriction and reductions in umbilical IGF1 concentrations [13,15], with no change in maternal IGF1. However, CSH RNAi results in two distinct pregnancy phenotypes [16]. The first is pregnancies with significant fetal and placental growth restriction (PI-FGR; [13,14,15]), and the second phenotype is represented by pregnancies with normal fetal and placental weights (non-FGR), even though there are significant changes in placental and fetal characteristics [17,18]. This disparity in CSH-deficient phenotypes, resulting from CSH RNAi, is supported by evidence in previous human studies [19,20]. We hypothesized that the impact of CSH RNAi could be mediated within the placenta by altering the expression of various components of the IGF axis and that the expression of the IGF axis within the CSH RNAi placenta may help explain the varying CSH RNAi phenotypes observed.

## 2. Materials and Methods

All animal procedures were approved by the Colorado State University Institutional Animal Care and Use Committee (Protocol #1576), the Institutional Biosafety Committee (18-092B), and the University of Colorado Anschutz Medical Campus Institutional Animal Care and Use Committee (Protocol #00714).

### 2.1. Experimental Groups

Two distinct cohorts of sheep were used as the basis of this study. The first cohort was previously reported by Tanner et al. [18] and consisted of 6 CSH RNAi pregnancies (2 males; 4 females) that did not exhibit fetal growth restriction, designated as CSH RNAi non-FGR, and only a tendency for restricted placental size, and a contemporary group of 6 NTS RNAi (non-targeting sequence controls) pregnancies (4 males; 2 females). The second cohort (not previously reported) consisted of 5 CSH RNAi pregnancies (3 males; 2 females) that exhibited significant reductions in both fetal and placental weights, comparable to the report of Baker et al. [13], designated as CSH RNAi PI-FGR, and 6 contemporary NTS RNAi pregnancies (3 males; 3 females).

### 2.2. Lentiviral Generation

Generation of hLL3.7-CSH (CSH RNAi) and hLL3.7 NTS (non-targeting sequence; control RNAi) constructs and lentivirus was previously described in detail by Baker et al. [13] and further discussed in Tanner et al. [16]. Briefly, both the CSH and NTS sequences were cloned into pLL3.7, and in so doing, the mouse U6 promoter was replaced with the human U6 promoter. Virus generation and titration were completed in accordance with Baker et al. [13]. The shRNA sequences for CSH and the NTS control are provided in Table 1.

### 2.3. Generation of CSH RNAi Pregnancies

All ewes (Dorper breed composition) were group housed in pens at the Colorado State University Animal Reproduction and Biotechnology Laboratory. They were provided access to hay, a trace mineral, and water to meet or slightly exceed their National Research Council requirements [21]. Animal management, estrus synchronization, and embryo transfers were all performed as previously described [13,16,18]. To summarize, after synchronization and breeding, expanded and hatched blastocysts were collected at 9 days gestational age (dGA) by flushing the uteri. Each blastocyst was then infected with either the NTS-control (hLL3.7 NTS shRNA) or CSH-targeting (hLL3.7 CSH shRNA) lentivirus. After a 5-h incubation period, each blastocyst was washed in media, and single blastocysts were surgically transferred into a synchronized recipient ewe. Each recipient ewe was monitored daily for return to standing estrus, and pregnancy was confirmed by ultrasound at 50 dGA.

### 2.4. Surgical Instrumentation of Fetus and Ewe

At approximately 115 dGA, pregnant recipient ewes were transported to the University of Colorado Anshutz Medical Campus, Perinatal Research Center (Aurora, CO, USA). All animals underwent surgical placement of fetal and maternal catheters to determine the effects of CSH RNAi on blood flow and nutrient transport as previously described [15,16,18]. Following catheterization (≈128 dGA), the ewes were given a minimum of 5 days to recover before the steady-state ^3^H_2_O transplacental diffusion technique was used to determine uterine and umbilical blood flows, and the repetitive sampling of maternal and fetal blood for the determination of uterine and umbilical uptakes of oxygen and glucose, as previously described [15,16,18]. Uterine vein and umbilical vein concentrations of CSH were determined by radioimmunoassay, as previously described [13,14,15,18,22]. Maternal (uterine) and fetal (umbilical) concentrations of insulin, IGF1, and cortisol were assessed by enzyme-linked immunosorbent assay (ALPCO Immunoassays 80-INSOV-E01, 22-IGFHU-E01, and 11-CORHU-E01-SLV, respectively), as described previously [23,24,25].

### 2.5. Tissue Collection

Immediately following the in vivo examination of these pregnancies (135 dGA), the ewe and fetus were euthanized with a lethal dose of pentobarbital (90 mg/kg; Fatal Plus, Vortech Pharmaceuticals, Dearborn, MI, USA) prior to tissue harvest. At necropsy, the gravid uterus (uteroplacental unit) was removed and weighed, the fetus was removed for measurement and organ harvest, all placentomes were trimmed from the endometrium, and the total number of placentomes and total placentome weight were recorded. Ten placentomes were randomly selected from each placenta and further separated into fetal cotyledons and maternal caruncles. The tissues were then snap-frozen in liquid nitrogen (N_2_) and stored at −80 °C.

### 2.6. RNA Isolation

Total cellular RNA was isolated from the maternal caruncles and fetal cotyledons using the RNeasy Mini Kit (Qiagen, Hilden, Germany) following the manufacturer’s protocol. RNA concentration was quantified using the BioTek Synergy 2 Microplate Reader and the Gen5 version 3.09 Microplate Reader and Imager Software (BioTek, Winooski, VT, USA). Quality was measured by the 260 to 280-nm absorbance ratio with a standard range of 2.0–2.3 requirement for all samples. Samples were then stored at −80 °C.

### 2.7. Complimentary DNA Synthesis and Quantitative Real-Time PCR (qPCR)

Complimentary DNA (cDNA) was synthesized from 2 μg of total cellular RNA using iScript Reverse Transcriptase Supermix fqPCR (BioRad Laboratories Inc., Hercules, CA, USA) according to the manufacturer’s protocol. An equal amount of cDNA (384 well plates: 2 uL/well) was used for each sample in the quantitative real-time PCR (qPCR) reaction. qPCR was performed using the CFX384 Real-Time System C1000 Thermal Cycler (BioRad Laboratories Inc.), and the protocol was followed as previously described [13]. All primer sets for qRT-PCR were designed using Oligo software (Molecular Biology Insight, Cascade, CO, USA) to amplify an intron-spanning product. Primer sequences and product sizes, as well as PCR conditions, are summarized in Table 2. A PCR product was generated for each gene using cDNA generated from 135 dGA fetal placenta (cotyledons) and cloned into the StrataClone vector (Agilent Technologies, Santa Clara, CA, USA). Each PCR product generated was sequenced (Colorado State University Proteomics and Metabolomics Facility) to verify the amplification of the correct cDNA. Standard curves were then generated for each mRNA, using the PCR products amplified from the sequenced plasmids, from 1 × 10^2^ to 1 × 10^−5^ pg, and were used to measure amplification efficiency. Starting quantities (pg) were normalized by dividing the starting quantity of mRNA of interest by the starting mRNA quantity (pg) of ribosomal S15 (*RPS15*), which has been previously determined not to be impacted by CSH RNAi [14].

### 2.8. Statistical Analysis

As the two separate CSH RNAi cohorts were not experimental contemporaries to each other because the pregnancies were generated and studied in different years, statistical comparisons were not made between the two cohorts. Furthermore, the pregnancy success rate limited the final number in each cohort, such that the study was not sufficiently powered to examine the effect of fetal sex. Accordingly, data derived from the respective cohort NTS RNAi and CSH RNAi pregnancies were statistically compared by unpaired Students *t*-test using GraphPad Prism (version 9) to analyze the main effects of treatment. Statistical significance was set at *p* ≤ 0.05 and a statistical tendency at *p* ≤ 0.10. Data are reported as the mean ± standard error of the mean (SEM). The data figures are presented as bar graphs representing the mean, and the capped vertical lines representing the SEM.

## 3. Results

### 3.1. Fetal and Placental Measurements

The fetal and placental measurements of the CSH RNAi non-FGR pregnancies were previously reported by Tanner et al. [18]. With the second cohort, the 5 CSH RNAi pregnancies exhibited fetal and placental weights that were ≥2 standard deviations below the mean of the NTS RNAi controls, similar to those reported by Baker et al. [13], and were designated as CSH RNAi PI-FGR pregnancies. As evidenced in Table 3, CSH RNAi PI-FGR pregnancies exhibited significant (*p* ≤ 0.05) reductions in fetal weight, fetal liver weight, crown-rump length, and placental weight, as well as reduced uterine and umbilical blood flow rates. Additionally, there were significant (*p* ≤ 0.05) reductions in uterine and umbilical uptakes of both glucose and oxygen (Table 3) and diminished uteroplacental utilization of both. While the umbilical uptake of both oxygen and glucose was significantly reduced (Table 3), umbilical vein (5.65 ± 0.14 vs. 4.97 ± 1.23 mmol/L; NTS RNAi vs. CSH RNAi PI-FGR; *p* = 0.22) and artery (3.85 ± 0.10 vs. 3.02 ± 0.83 mmol/L; *p* = 0.11) oxygen content was not statistically impacted, whereas umbilical vein (1.20 ± 0.03 vs. 0.77 ± 0.20 mmol/L; NTS RNAi vs. CSH RNAi PI-FGR; *p* = 0.002) and artery (1.02 ± 0.04 vs. 0.66 ± 0.12 mmol/L; *p* = 0.008) glucose concentrations were both significantly reduced.

### 3.2. 135 dGA Plasma Hormone Concentrations

For the CSH RNAi PI-FGR pregnancies, maternal CSH was significantly (*p* ≤ 0.05) depressed (Table 4), whereas maternal concentrations of insulin, IGF1, and cortisol were not impacted by CSH RNAi. By contrast, umbilical concentrations of both insulin and IGF1 were significantly (*p* ≤ 0.05) depressed (Table 4) in CSH RNAi PI-FGR pregnancies, umbilical cortisol concentrations were significantly (*p* ≤ 0.05) elevated, and umbilical CSH concentration was not impacted.

### 3.3. 135 dGA Placental IGF’s, IGFBP’s, and IGFR’s mRNA Concentrations

**Figure 1:**
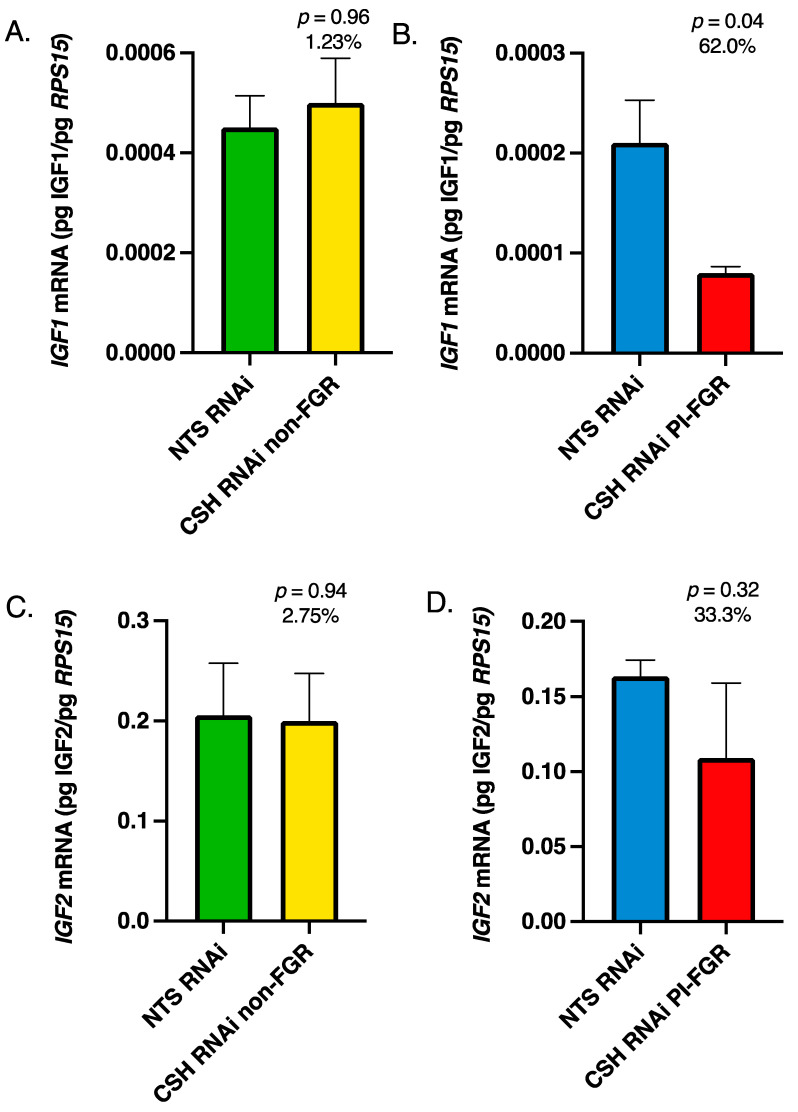
135 dGA fetal cotyledon tissue (**A**) non-FGR *IGF1* mRNA concentrations, (**B**) PI-FGR *IGF1* mRNA concentrations, (**C**) non-FGR *IGF2* mRNA concentrations, and (**D**) PI-FGR *IGF2* mRNA concentrations. Data are shown as means ± SEM for the starting quantity of the mRNA of interest (pg) divided by the starting quantity (pg) of the housekeeping mRNA of *RPS15*.

**Figure 2:**
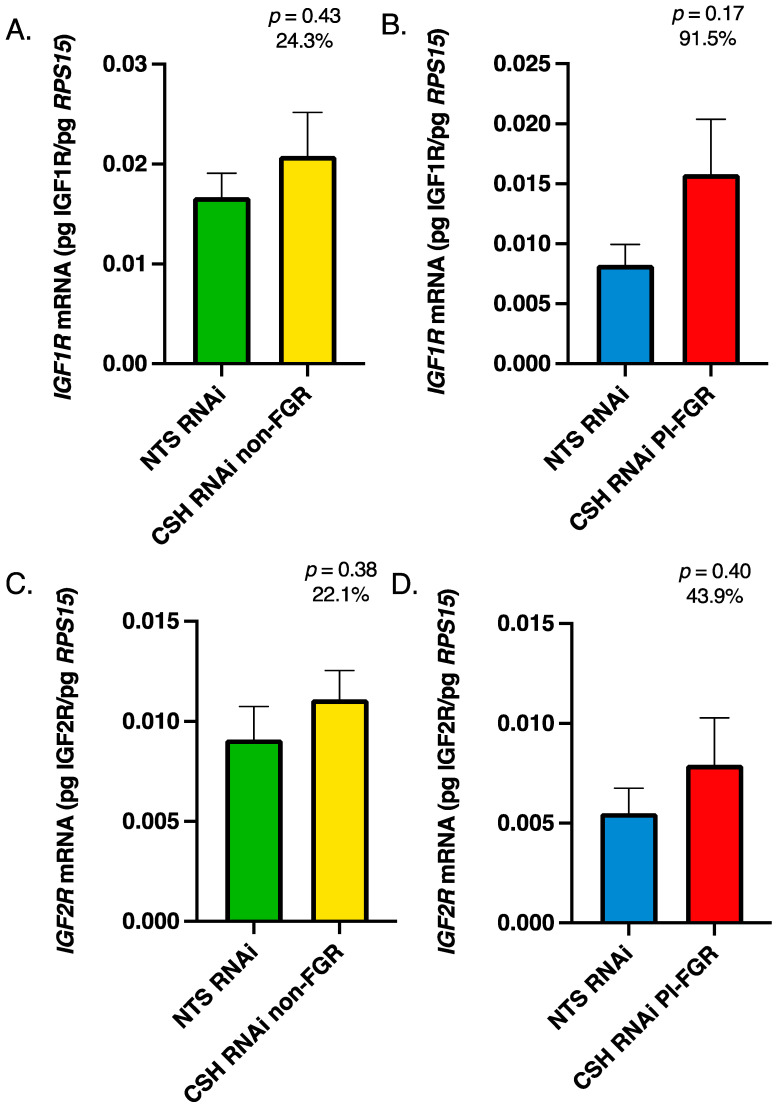
135 dGA fetal cotyledon tissue (**A**) non-FGR *IGF1R* mRNA concentrations, (**B**) PI-FGR *IGF1R* mRNA concentrations, (**C**) non-FGR *IGF2R* mRNA concentrations, and (**D**) PI-FGR *IGF2R* mRNA concentrations. Data are shown as means ± SEM for the starting quantity of the mRNA of interest (pg) divided by the starting quantity (pg) of the housekeeping mRNA of *RPS15*.

Fetal cotyledon and maternal caruncle mRNA harvested at 135 dGA were subjected to qPCR to determine *IGF1*, *IGF2*, *IGFBP1-3*, *IGF1R,* and *IGF2R* mRNA concentrations. While the CSH RNAi non-FGR pregnancies did not differ in fetal cotyledon *IGF1* or *IGF2* mRNA concentrations (Figure 1A,C), the CSH RNAi PI-FGR pregnancies exhibited reduced *IGF1* mRNA (*p* < 0.05; Figure 1B). While *IGF2* mRNA was not significantly impacted by treatment in the CSH RNAi PI-FGR pregnancies, it was reduced by 33.3% (Figure 1D). The mRNA concentration for *IGF1R* and *IGF2R* did not differ due to treatment in the non-FGR pregnancies (Figure 2A,C). In the CSH RNAi PI-FGR pregnancies, neither *IGF1R* (Figure 2B) or *IGF2R* (Figure 2D) mRNA were statistically impacted by treatment, although *IGF1R* mRNA concentration was 91.5% greater (*p* = 0.17).

**Figure 3:**
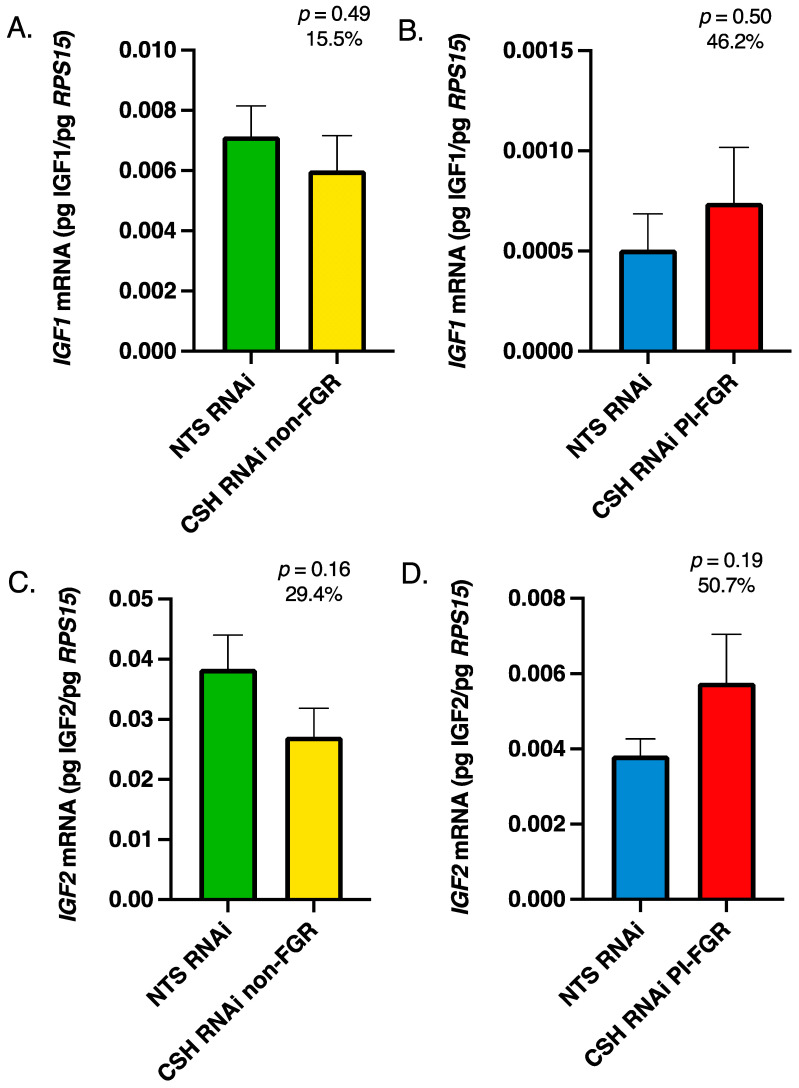
135 dGA maternal caruncle tissue (**A**) non-FGR *IGF1* mRNA concentrations, (**B**) PI-FGR *IGF1* mRNA concentrations, (**C**) non-FGR *IGF2* mRNA concentrations, and (**D**) PI-FGR *IGF2* mRNA concentrations. Data are shown as means ± SEM for the starting quantity of the mRNA of interest (pg) divided by the starting quantity (pg) of the housekeeping mRNA of *RPS15*.

**Figure 4:**
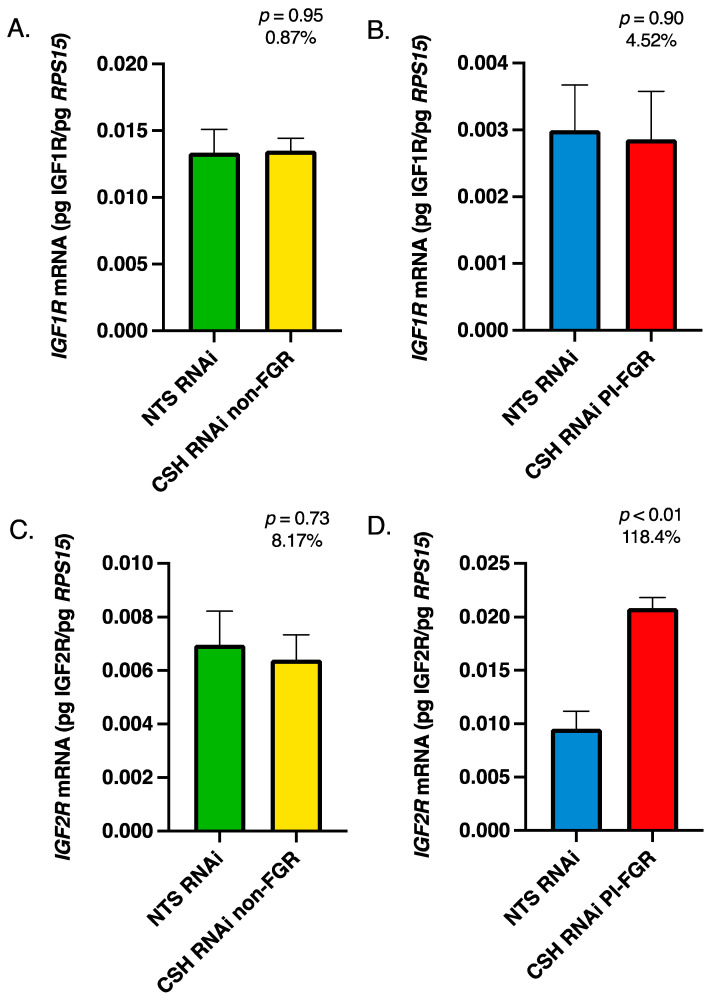
135 dGA maternal caruncle tissue (**A**) non-FGR *IGF1R* mRNA concentrations, (**B**) PI-FGR *IGF1R* mRNA concentrations, (**C**) non-FGR *IGF2R* mRNA concentrations, and (**D**) PI-FGR *IGF2R* mRNA concentrations. Data are shown as means ± SEM for the starting quantity of the mRNA of interest (pg) divided by the starting quantity (pg) of the housekeeping mRNA of *RPS15*.

Within the maternal portion of the placenta (caruncles), there were no significant changes in *IGF1* or *IGF2* mRNA concentrations in either the CSH RNAi non-FGR or PI-FGR pregnancies (Figure 3). Maternal caruncle *IGF1R* mRNA was not impacted by CSH RNAi in either non-FGR or PI-FGR pregnancies (Figure 4A,B, respectively) nor was *IGF2R* mRNA impacted in CSH RNAi non-FGR pregnancies (Figure 4C). However, *IGF2R* mRNA concentration was significantly increased in the maternal caruncles of CSH RNAi PI-FGR pregnancies (Figure 4D).

**Figure 5:**
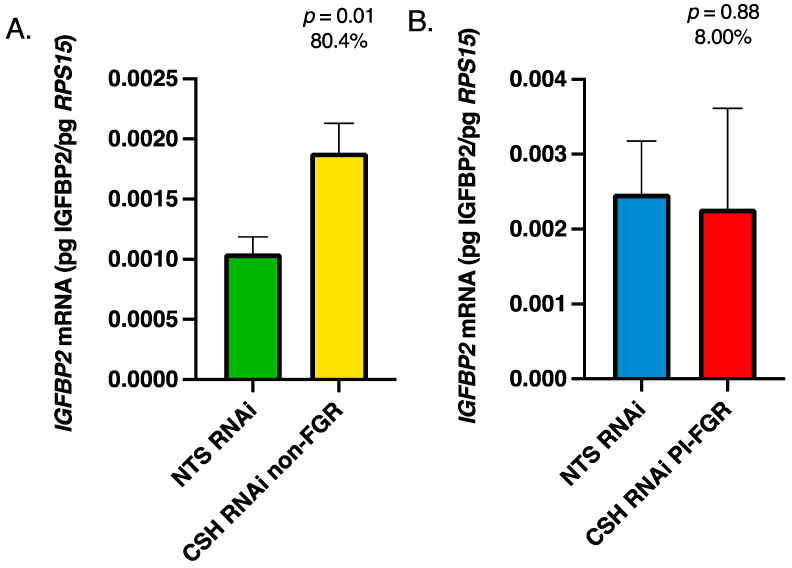
135 dGA fetal cotyledon tissue (**A**) non-FGR *IGFBP2* mRNA concentrations, (**B**) PI-FGR *IGFBP2* mRNA concentrations. Data are shown as means ± SEM for the starting quantity of the mRNA of interest (pg) divided by the starting quantity (pg) of the housekeeping mRNA of *RPS15*.

**Figure 6:**
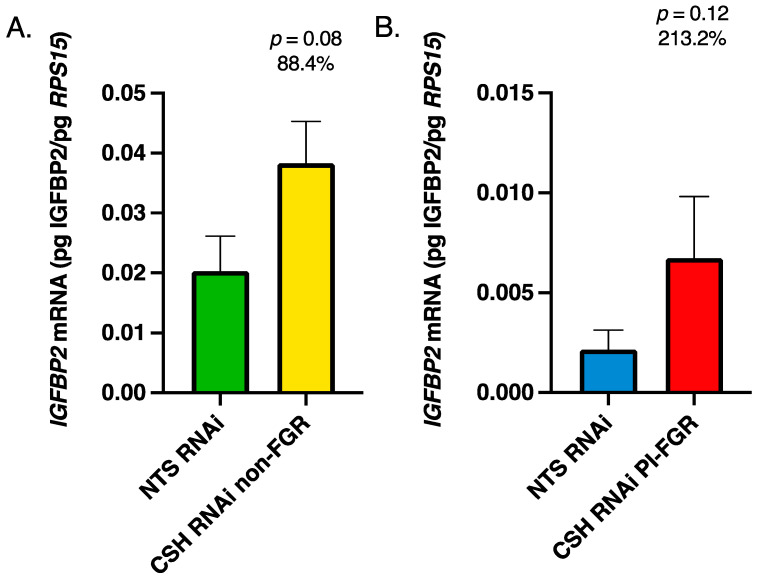
135 dGA maternal caruncle tissue (**A**) non-FGR *IGFBP2* mRNA concentrations, (**B**) PI-FGR *IGFBP2* mRNA concentrations. Data are shown as means ± SEM for the starting quantity of the mRNA of interest (pg) divided by the starting quantity (pg) of the housekeeping mRNA of *RPS15*.

There were no significant changes in the mRNA concentrations of either *IGFBP1* or *IGFBP3* in either the fetal cotyledons or maternal caruncles because of CSH RNAi (data not presented). However, as evidenced in Figure 5A, *IGFBP2* mRNA concentration was significantly increased in the fetal cotyledons of CSH RNAi non-FGR placenta, whereas there was no effect of CSH RNAi on PI-FGR concentrations of *IGFBP2* mRNA (Figure 5B). In the maternal portion (caruncles) of the placenta, there was a statistical tendency (*p* ≤ 0.10) for *IGFBP2* mRNA concentration to be increased in CSH RNAi non-FGR placenta (Figure 6A) and a similar tendency in CSH RNAi PI-FGR maternal caruncles (Figure 6B).

## 4. Discussion

Placental development and function are prerequisites for fetal growth, and it has been reported [26] that 60% of FGR pregnancies with a normally formed fetus result from altered placental function. The placenta is not only responsible for the uptake of maternal nutrients and the partial transfer of these nutrients to the fetus but is also a source of specific hormones that can impact maternal and fetal physiology. One such placental hormone is chorionic somatotropin/placental lactogen (CSH/PL), which has long been hypothesized to directly affect maternal and fetal metabolism and endocrine function [12]. However, direct evidence supporting this hypothesis was not reported until Baker et al. [13]. Using in vivo lentiviral-mediated RNAi, they demonstrated that CSH RNAi resulted in significant fetal and placental growth restriction (PI-FGR) near term. In the report of Baker et al. [13], there were two unexpected results. The first was the large impact on placental mass, as a role for CSH augmenting placental growth had not been hypothesized previously. The second was that some of the pregnancies harvested near-term appeared to be normal, and the remainder exhibited placental and fetal weights ≥2 SD below the mean of the control pregnancies. However, further analysis of these [13] non-FGR pregnancies by Ali et al. [17] revealed that there were significant differences with the non-FGR fetuses, even when birthweight was not impacted. The dichotomy in late-term phenotype observed with CSH RNAi is supported by case reports of human pregnancies in which deletions or mutations in the *CSH* loci resulted in FGR or apparently normal outcomes [27,28,29,30,31,32]. This is supported by the results of Daikoku et al. [19] and Lindberg and Nilsson [20], which observed both normal birth weights and FGR in pregnancies with suppressed maternal concentrations of CSH. Based on the observations of Ali et al. [17], this raises the question of how “normal” the outcomes of human pregnancies deficient in CSH without FGR are.

Herein, we report the impact of CSH RNAi on the expression of the IGF axis in both the maternal and fetal components of the placenta from a cohort of CSH RNAi non-FGR pregnancies [18] as well as a cohort of CSH RNAi PI-FGR pregnancies in which the impact on placental and fetal growth was very similar to that originally reported by Baker et al. [13]. With both CSH RNAi cohorts, the pregnancies underwent maternal and fetal cannulation, allowing the direct assessment of uterine and umbilical blood flows, uterine uptake of nutrients, uteroplacental nutrient utilization, and umbilical nutrient uptakes under non-stressed, non-anesthetized conditions [16,18]. As previously reported by Tanner et al. [18], the primary difference in CSH RNAi non-FGR pregnancies and the NTS RNAi controls was the significant reductions in uterine blood flow, coupled with increased uteroplacental utilization of glucose, resulting in the fraction of glucose taken up by the uterus and utilized by the fetus being significantly reduced. By contrast, the CSH RNAi PI-FGR pregnancies (Table 3) exhibited an even greater difference in uterine blood flow and a significant reduction in umbilical blood flow. Furthermore, the CSH RNAi PI-FGR pregnancies had significant reductions in uterine uptake, uteroplacental utilization, and umbilical uptake of both oxygen and glucose, resulting in significant reductions in fetal and placental weights (Table 3). Comparing these two CSH RNAi phenotypes suggests that a common consequence resulting from CSH RNAi is significantly reduced uterine blood flow, and the magnitude of this reduction in uterine blood flow may determine the degree by which placental and fetal weights are impacted as a result of limiting nutrient uptake and transfer.

With the CSH RNAi PI-FGR pregnancies, uterine artery insulin, IGF1, and cortisol concentrations were not impacted, whereas umbilical concentrations of insulin and IGF1 were significantly reduced (Table 4), and umbilical artery cortisol was significantly increased. The impact of CSH RNAi in PI-FGR pregnancies on umbilical insulin, IGF1, and cortisol is similar to that reported by Tanner et al. [15]. Significant reductions in umbilical insulin and IGF1 are often observed in a variety of sheep models of FGR [33,34]. Elevated umbilical cortisol concentrations during late gestation have also been observed during late gestation in FGR sheep pregnancies. They can be viewed as a fetal stress response, even in response to short-term fasting [35], and glucocorticoid administration can induce fetal metabolic changes observed with FGR [34,36].

The reduction in fetal cotyledon tissue CSH concentration resulting from CSH RNAi has been fairly consistent across studies [13,15,18], but the impact on uterine vein and umbilical vein CSH concentrations has been more variable. With the original report of Baker et al. [13], only a 24% reduction in uterine vein CSH concentration was observed, which did not reflect the greater reduction in fetal cotyledon tissue concentrations of *CSH* mRNA or CSH. By contrast, in the current study, we report a ≈56% reduction in uterine vein CSH concentration within the CSH RNAi PI-FGR pregnancies, which exhibited similar phenotypic changes to those reported by Baker et al. [13]. A major difference between these two studies is that the Baker et al. [13] samples were collected during a terminal surgery rather than following cannulation and took numerous days to recover. Taylor et al. [37] reported elevated concentrations of CSH for several days following surgery, and therefore collecting samples during a terminal surgery could “artificially” elevate CSH concentrations, potentially masking the impact of CSH RNAi, such that the current data collected under non-anesthetized non-stressed conditions is more reflective of the impact of CSH RNAi on maternal CSH concentrations.

In contrast to the impact on uterine vein concentrations of CSH, none of the CSH RNAi studies [13,15,18], including the current CSH RNAi PI-FGR pregnancies (Table 4), show a difference in umbilical vein CSH concentrations. One possible explanation for this is that any reduction in the CSH entry rate into the umbilical circulation [38] may have been masked by reductions in fetal size and blood volume. Another likely explanation for this resides with the fetal hypoglycemia reported [15] and observed in the current study. Freemark et al. [39] reported that fasting-induced fetal hypoglycemia resulted in significant elevations in umbilical CSH concentrations, which were “normalized” upon maternal glucose infusion. Recently, Lynch et al. [40] reported that the fetal hypoglycemia observed at mid-gestation as a result of fetal placental SLC2A3 RNAi was associated with a 70% increase in umbilical CSH concentrations, with no impact on uterine vein concentrations. As such, the fetal hypoglycemia resulting from CSH RNAi may have subsequently induced a relative elevation in umbilical CSH concentrations, potentially overcoming the deficit in CSH being secreted into the umbilical circulation as a result of CSH RNAi. Regardless of the exact explanation for these data, they highlight the complexity of the interactions amongst the three functional compartments (maternal, placental, and fetal) of pregnancy [41] and the need to investigate all three simultaneously.

Following the initial report of Baker et al. [13], Jeckel et al. [14] examined the mRNA concentrations of the IGF axis in the fetal cotyledon portion of the placenta from those pregnancies reported in Baker et al. [13]. At 135 dGA, Jeckel et al. [14] reported significant reductions in fetal placenta *IGF1* and *IGF2* mRNA concentrations, with no significant changes in *IGFBP1*, *IGFBP2*, and *IGFBP3* mRNA concentrations. Jeckel et al. [14] did not assess *IGF1R* and *IGF2R* mRNA concentrations, nor did they assess IGF axis expression in the maternal placenta (caruncles) or either placental tissue derived from the non-FGR pregnancies reported by Baker et al. [13]. This led us to examine the IGF axis in both the maternal and fetal placenta and from CSH RNAi non-FGR and PI-FGR pregnancies. Similar to what was reported by Jeckel et al. [14], we observed a significant reduction in fetal placenta (cotyledon) *IGF1* mRNA concentration in the CSH RNAi PI-FGR pregnancies. However, no difference in *IGF1* mRNA concentration was identified in the maternal placenta (caruncle) of the CSH RNAi PI-FGR pregnancies. Furthermore, *IGF1* mRNA concentration was not impacted by CSH RNAi in either the maternal or fetal placenta of non-FGR pregnancies. As reported herein (Table 4) and previously [13,15], umbilical artery concentrations of IGF1 are significantly reduced in CSH RNAi PI-FGR pregnancies, which is associated with significantly smaller fetal livers and reduced concentrations of fetal liver *IGF1* mRNA concentrations. Infusion of CSH into fetal sheep for 14 days results in significantly increased fetal arterial concentrations of IGF1 [42]. Therefore, the reduction in fetal placenta *IGF1* mRNA in CSH RNAi PI-FGR pregnancies may well result from a lack of intra-placental CSH stimulation of *IGF1* transcription.

IGF1 has been shown to enhance the expression of the facilitative glucose transporter SLC2A1 in human trophoblast cells [43] and the mouse placenta [44]. In sheep, FGR induced by placental embolization [45] results in fetal hypoxia and hypoglycemia and is associated with reductions in *SLC2A1* mRNA and placental glucose uptake, but the intra-amniotic infusion of IGF1 into these fetuses increased the placental expression of *SLC2A1* mRNA [45]. The reduction in fetal placental *IGF1* mRNA, and presumably placental IGF1, may have played a role in the reduction in uterine and umbilical glucose uptake with the CSH RNAi PI-FGR pregnancies. However, Tanner et al. [18] reported a 25% reduction (*p* ≤ 0.10) in fetal placenta SLC2A1 protein concentration in the CSH RNAi non-FGR pregnancies, which did not exhibit a reduction in fetal placenta *IGF1* mRNA (Figure 1A). There appears to be a reciprocal relationship between IGF1 and glucose since the infusion of fetal sheep with glucose results in elevated fetal concentrations of IGF1 [46], such that the reduction in fetal placental *IGF1* mRNA may have been impacted by reduced uterine uptake and uteroplacental utilization of glucose in the CSH RNAi PI-FGR pregnancies. Beyond the interplay of glucose and IGF1, CSH is significantly elevated in fetal circulation as a result of fetal hypoglycemia [39,40]. However, the increased umbilical CSH as a result of fetal hypoglycemia was associated with the maintenance of fetal concentrations of IGF1. Consequently, the interplay of CSH, IGF1, and glucose between the three compartments of pregnancy may ultimately impact the degree of placental and fetal growth restriction that occurs in FGR pregnancies.

In contrast to what was reported by Jeckel et al. [14], there were no significant changes in *IGF2* mRNA concentrations within the fetal placenta in either the CSH RNAi non-FGR or PI-FGR pregnancies. In the PI-FGR pregnancies, there was a 33% reduction in *IGF2* mRNA, but this was not statistically different, whereas Jeckel et al. [14] reported a statistically significant 53% reduction in *IGF2* mRNA. The characteristics of the current CSH RNAi PI-FGR pregnancies and those from which the Jeckel et al. [14] samples were derived are very similar, except for sample collection methods. The fetal cotyledons examined by Jeckel et al. [14] were collected following an 18 h fast and anesthesia [13], which may have impacted *IGF2* mRNA concentrations. In another sheep model of PI-FGR [4], *IGF2* mRNA was significantly increased in the fetal placenta at 55 dGA but not at 90 dGA, and near-term data was not reported. Interestingly, *IGF1* mRNA in the fetal placenta was not impacted at either gestational age, possibly indicating that IGF2 plays a predominant role in early placental development, with IGF1 being more important during late gestation. In mice, placenta-specific *Igf2* gene ablation [7] clearly demonstrated the importance of Igf2 in rodent placental growth and function. Since the rat placenta is thought to not express *Igf1* [47], and *Igf1* gene ablation in mice did not impact placenta size [3], it appears that in rodents, Igf2 drives placental growth and function rather than Igf1. However, it is possible that IGF1 plays the predominant role in non-rodent placental growth and function, especially during later gestation. Within the maternal placenta (caruncles), *IGF2* mRNA concentrations were not statistically different in either the CSH RNAi non-FGR or PI-FGR pregnancies, although *IGF2* mRNA was 29% lower in non-FGR pregnancies and 51% greater in PI-FGR pregnancies (Figure 3). The lack of a significant effect of CSH RNAi on *IGF2* mRNA within the maternal caruncles agrees with a lack of effect at either 55 or 90 dGA in the pregnancies reported by de Vrijer et al. [4].

While there are two IGF receptors, IGF1R and IGF2R, both IGF1 and IGF2 activate IGF1R [5], resulting in enhanced cell proliferation, differentiation, and/or migration [48,49]. In contrast, IGF2R primarily modulates the availability of IGF2 [5] and acts as a clearance receptor for IGF2 [50]. Within the CSH RNAi non-FGR pregnancies, there were no significant changes in *IGF1R* or *IGF2R* mRNA concentrations in either the fetal (Figure 2) or maternal (Figure 4) components of the placenta. However, in the fetal placenta of CSH RNAi PI-FGR pregnancies, *IGF1R* mRNA increased by 92%, although this did not reach statistical significance (Figure 2), which may have been an attempt to upregulate IGF1R in the face of significantly reduced *IGF1* mRNA in the fetal placenta (Figure 1). In the maternal placenta of CSH RNAi PI-FGR pregnancies, neither the concentrations of *IGF1* or *IGF1R* mRNA were impacted, collectively suggesting that IGF1 did not play a role in the maternal placenta response (e.g., reduced uterine blood flow, etc.) to CSH RNAi in either the non-FGR and PI-FGR pregnancies. Interestingly, the concentration of *IGF2R* mRNA was significantly elevated (118%; Figure 4) within the maternal placenta of CSH RNAi PI-FGR pregnancies, potentially to offset the effects of any increased production of IGF2 by the maternal placenta (Figure 3). One possible explanation for the increased *IGF2R* mRNA in CSH RNAi PI-FGR pregnancies is a response to the significant reduction in uterine uptake of glucose. When fetal placenta glucose uptake is diminished in response to SLC2A3 RNAi [40], similar increases in *IGF2* and *IGF2R* mRNA concentrations were reported as to what was observed in the current CSH RNAi PI-FGR maternal placenta. The physiological importance of this finding is not at all clear but may suggest the need to maintain a fine balance between IGF1 and IGF2 and the actions they propagate through IGFR1, thereby inhibiting the autocrine and paracrine actions of IGF2 mediated through IGF1R [51,52,53].

The IGFBPs can potentiate or inhibit the actions of IGFs, dependent on the binding protein’s preference for IGF1 and IGF2 [54,55]. Similar to what was reported by Jeckel et al. [14], we did not observe any significant differences in the concentration of *IGFBP1* or *IGFBP3* mRNA in either the fetal or maternal placenta, in either the CSH RNAi non-FGR or PI-FGR pregnancies. However, in the CSH RNAi non-FGR pregnancies, the concentration of *IGFBP2* mRNA was significantly increased in both the fetal and maternal portions of the placenta (Figure 5 and Figure 6) and a non-statistically significant increase within the maternal caruncles of CSH RNAi PI-FGR pregnancies. IGFBP2 is reported to inhibit IGF actions, particularly IGF2, as it has a 2-fold higher affinity for IGF2 [56,57]. When IGFBP2 is overexpressed in human embryonic kidney fibroblasts, cell proliferation is inhibited and is reversed with the addition of IGFs [58]. However, other studies report that IGFBP2 promoted tumor cell proliferation [59], likely in an IGF-independent manner [60]. In the pig, the uterine glandular epithelium co-express IGF2 and IGFPB2, and Badinga et al. [61] demonstrated both IGF2-dependent and independent effects of IGFBP2 on glandular epithelial cell proliferation. The enhanced concentration of *IGFBP2* mRNA within the CSH RNAi non-FGR pregnancies may be one mechanism by which uterine and placental growth was maintained in these pregnancies. Since *IGFBP2* mRNA concentrations were also elevated in the maternal caruncles of CSH RNAi PI-FGR pregnancies, this might implicate the significantly reduced uterine blood flow that occurs in both the non-FGR and PI-FGR pregnancies as a potential driver of enhanced *IGFBP2* expression.

## 5. Conclusions

Investigating the interplay of the three compartments of pregnancy is complex, but it is necessary to understand the progression of normal pregnancies and determine the etiology of pregnancy complications such as FGR. By combining in vivo lentiviral-mediated RNAi with in vivo steady-state assessment of blood flows, as well as nutrient uptakes and utilization by both maternal and fetal tissues, we were able to compare CSH RNAi pregnancies that do not result in FGR and those that result in PI-FGR. Clearly, nutrient uptakes, utilization, and hormone secretion are greatly impacted in PI-FGR pregnancies, but non-FGR pregnancies are not truly “normal.” This also allowed us to examine the mRNA concentrations of the IGF axis in both the maternal (caruncles) and fetal (cotyledons) placenta. As might be expected, the majority of differences observed were within the PI-FGR pregnancies, especially the impact on fetal placental *IGF1* mRNA, which fits with CSH RNAi’s impacts on the fetal liver and umbilical concentrations of IGF1. However, in non-FGR pregnancies, enhanced *IGFBP2* mRNA within both maternal and fetal placenta may suggest a compensatory mechanism that maintains uterine and placental growth. This potentially IGF-independent action of IGFBP2 to promote placental growth needs further investigation, along with the potential role of reduced uterine blood flow inducing *IGFBP2* mRNA.

## Figures and Tables

**Table 1:** Control and CSH-targeting short-hairpin RNA (shRNA) sequences.

Oligonucleotide	Sequence (5′–3′)
Control (NTS) shRNA sense	GAGTTAAAGGTTCGGCACGAATTCAAGAGATTCGTGCCGAACCTTTAACTC
CSH-targeting shRNA sense	AAGGCCAAAGTACTTGTAGACTTCAAGAGAGTCTACAAGTACTTTGGCCTT

**Table 2:** PCR primers, annealing temperatures, and product sizes for qPCR.

cDNA	Forward Primer (5′–3′)	Reverse Primer (5′–3′)	Anneal, °C	Product, bp
*RPS15*	atcattctgcccgagatggtg	tgctttacgggcttgtaggtg	58	124
*IGF1*	tcgcatctctcttctatctggccctgt	acagtacatctccagcctcctcaga	62	238
*IGF2*	gaccgcggcttctacttcag	aagaacttgcccacggggtat	62	202
*IGFBP1*	tgatgaccgagtccagtgag	gtccagcgaagtctcacac	62	247
*IGFBP2*	caatggcgaggagcactctg	tggggatgtgtagggaatag	55	330
*IGFBP3*	ctcagagcacagacaccca	ggcatatttgagctccac	54	335
*IGF1R*	aactgtcatctccaacctc	caagcctcccactatcaac	60	493
*IGF2R*	gacttgtgtccagaccagattc	gccgtcgtcctcactctcatc	58	674

*RPS15*, ribosomal protein S15; *IGF*, insulin-like growth factor; *IGFBP*, IGF-binding protein; *IGF1R* and *IGF2R*, Type 1 and Type 2 IGF receptor, respectively.

**Table 3:** Measurements collected at necropsy 135 dGA for CSH RNAi PI-FGR pregnancies.

Variable	Experimental Group		*p*-Value	% Change
	NTS RNAi	CSH RNAi PI-FGR		
Fetal weight, g	4625.6 ± 37.1	3226.4 ± 325.8	≤0.01	30.2
Fetal liver weight, g	125.2 ± 3.32	74.2 ± 9.04	≤0.01	40.7
Crown-rump length, cm	51.4 ± 0.71	46.9 ± 1.37	0.02	8.76
Placental weight, g	517.7 ± 28.6	247.9 ± 20.5	≤0.01	52.1
Total placentome number	70.5 ± 5.86	58.0 ± 1.79	0.09	17.7
Uterine blood flow, mL/min	2477.6 ± 501.9	1453.7 ±403.9	0.03	41.3
Umbilical blood flow, mL/min	854.6 ± 41.3	496.9 ± 170.1	≤0.01	41.9
Uterine glucose uptake, μmol/min	423.0 ± 84.6	266.9 ± 75.2	0.04	36.9
Umbilical glucose uptake, μmol/min	137.5 ± 9.85	78.4 ± 28.5	0.02	43.0
Uteroplacental glucose utilization, μmol/min	292.7 ± 62.0	188.1 ± 75.2	0.17	35.7
Uterine oxygen uptake, mmol/min	3.01 ± 0.56	1.79 ± 0.49	≤0.01	40.3
Umbilical oxygen uptake, mmol/min	1.53 ± 0.09	0.93 ± 0.33	0.02	39.5
Uteroplacental oxygen utilization, mmol/min	1.53 ± 0.30	0.94 ± 0.35	0.06	38.4

Data are shown as mean values ± SEM for all pregnancies in each treatment group. NTS RNAi = non-targeting sequence RNAi controls; CSH RNAi PI-FGR = CSH RNAi resulting in placental insufficiency (PI) and fetal growth restriction (FGR).

**Table 4:** 135 dGA Plasma hormone concentrations in CSH RNAi PI-IUGR pregnancies.

Variable	Experimental Group		*p*-Value	% Change
	NTS RNAi	CSH RNAi PI-FGR		
Uterine Vein CSH, ng/mL	371.2 ± 64.6	165.3 ± 49.5	0.05	55.5
Umbilical Vein CSH, ng/mL	32.0 ± 3.28	31.1 ± 6.88	0.89	2.86
Uterine Artery Insulin, ng/mL	0.54 ± 0.14	0.67 ± 0.19	0.60	22.5
Umbilical Artery Insulin, ng/mL	0.73 ± 0.10	0.35 ± 0.08	0.02	52.9
Uterine Artery IGF1, ng/mL	170.7 ± 13.8	151.9 ± 14.2	0.39	11.0
Umbilical Artery IGF1, ng/mL	167.5 ± 23.8	72.0 ± 20.13	0.02	57.0
Uterine Artery Cortisol, ng/mL	159.5 ± 62.2	121.3 ± 59.7	0.67	23.9
Umbilical Artery Cortisol, ng/mL	51.4 ± 4.66	129.2 ± 28.0	0.01	151.5

Data are shown as mean values ± SEM for each treatment group. NTS RNAi = non-targeting sequence RNAi controls; CSH RNAi PI-FGR = CSH RNAi resulting in placental insufficiency (PI) and fetal growth restriction (FGR).

## Data Availability

Data are available upon request to the corresponding author.
